# Platelet Granule Exocytosis: A Comparison with Chromaffin Cells

**DOI:** 10.3389/fendo.2013.00077

**Published:** 2013-06-26

**Authors:** Jennifer L. Fitch-Tewfik, Robert Flaumenhaft

**Affiliations:** ^1^Division of Hemostasis and Thrombosis, Department of Medicine, BIDMC, Harvard Medical School, Boston, MA, USA

**Keywords:** exocytosis, granules, platelets, chromaffin system, cytoskeleton, dynamins, SNAREs

## Abstract

The rapid secretion of bioactive amines from chromaffin cells constitutes an important component of the fight or flight response of mammals to stress. Platelets respond to stresses within the vasculature by rapidly secreting cargo at sites of injury, inflammation, or infection. Although chromaffin cells derive from the neural crest and platelets from bone marrow megakaryocytes, both have evolved a heterogeneous assemblage of granule types and a mechanism for efficient release. This article will provide an overview of granule formation and exocytosis in platelets with an emphasis on areas in which the study of chromaffin cells has influenced that of platelets and on similarities between the two secretory systems. Commonalities include the use of transporters to concentrate bioactive amines and other cargos into granules, the role of cytoskeletal remodeling in granule exocytosis, and the use of granules to provide membrane for cytoplasmic projections. The SNAREs and SNARE accessory proteins used by each cell type will also be considered. Finally, we will discuss the newly appreciated role of dynamin family proteins in regulated fusion pore formation. This evaluation of the comparative cell biology of regulated exocytosis in platelets and chromaffin cells demonstrates a convergence of mechanisms between two disparate cell types both tasked with responding rapidly to physiological stimuli.

## Introduction

**Figure 1:**
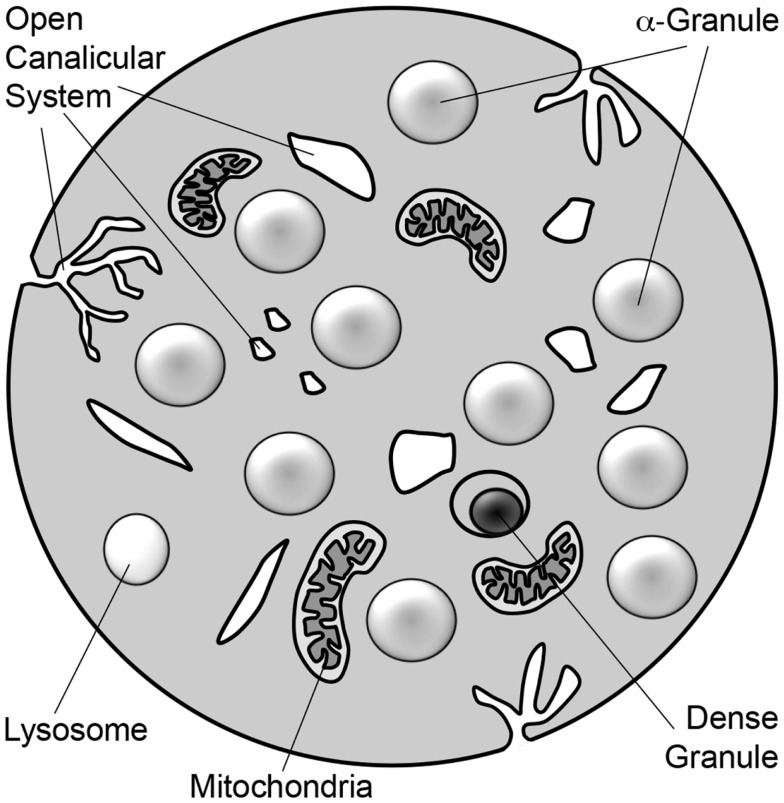
**Schematic diagram of platelet**. The platelet is a 2–3 μm discoid cell that contains α-granules, dense granules, and lysosomes. Platelets also contain mitochondria. Tunnel invaginations of the plasma membrane forms a complex membrane network, termed the open canalicular system, that courses throughout the platelet interior. Platelet granule secretion is thought to occur through fusion of granules with either the plasma membrane or the open canalicular system.

Platelets are small, anucleate blood cells derived from bone marrow megakaryocytes. They are best known for their central role in maintaining the integrity of the vasculature (hemostasis) and for their pathological role in clotting arteries and veins (thrombosis) during myocardial infarction, stroke, peripheral vascular disease, and deep vein thrombosis. In addition to their role in hemostasis, platelets have also been proposed to function in many other aspects of host defense. Stimulus-induced release of platelet granules contributes to nearly all platelet functions including hemostasis and thrombosis, inflammation, angiogenesis, and anti-microbial activities (Blair and Flaumenhaft, 2009). Platelets contain three granule types: α-granules, dense granules, and lysosomes (Figure 1; Table 1). Absence of dense granules, as observed in inherited syndromes such as Hermansky–Pudlak syndrome or Chediak–Higashi syndrome, results in a bleeding diathesis (Hermansky and Pudlak, 1959). Absence of α-granules, as observed in gray platelet syndrome, also increases bleeding (Buchanan and Handin, 1976; Costa et al., 1976). The bleeding phenotype associated with these disorders underscores the importance of platelet granules in hemostasis.

**Table 1:** **Comparison of platelets and chromaffin cells**.

	Platelets	Chromaffin cells
**Distribution**	Intravascular	Adrenal medulla
**Size**	2–3 μm	∼20 μm
**Functions**	Hemostasis/thrombosis	Blood pressure modulation
	Inflammation	Paracrine signaling
	Angiogenesis	Anti-microbial host defense
	Anti-microbial host defense	Immune regulation
	Mitogenesis	Analgesia
**Granule types**	α-Granules, dense granules, and lysosomes	Large dense-core vesicles (LDCVs) and synaptic-like microvesicles (SLMVs)

Despite the functional importance of platelet granule secretion in maintaining vascular integrity and promoting host defense, the molecular basis of platelet granule secretion remained poorly studied until the late 1990s, despite transformative advances in secretion biology that had occurred over the preceding decade (Rothman and Orci, 1992; Sollner et al., 1993). This knowledge deficit was due in part to the fact that platelets are anucleate, complicating the use of standard molecular biological approaches that have been widely used to study regulated secretion in nucleated cells. In addition, the small size (2–3 μm in diameter) and unusual membrane system of the platelet prevented application of classic electrophysical approaches such as patch-clamp studies. Earlier studies evaluating the molecular mechanisms of platelet granule secretion relied on applying knowledge derived from other systems to the study of platelets. The chromaffin cell has been influential in this regard. Although these two cell types have different embryonic derivations and functions, both cells store bioactive amines and peptides at high concentrations and release their cargos rapidly in response to stress signals (Table 1). The study of platelet granule secretion has matured considerably over the past decade, making relevant a comparison of the mechanisms by which platelets and chromaffin cells store and release their granule contents in response to environmental signals.

## Platelet Granule Types

### α-Granules

α-Granules are by far the most abundant platelet granule type (Figure 1). There are ∼50–80 α-granules/platelet, ranging in size from 200 to 500 nm. They comprise roughly 10% of the platelet volume, 10-fold more than dense granules. α-Granules contain a variety of membrane proteins and soluble cargo that give them a distinct appearance when stained with osmium and viewed by transmission electron microscopy (TEM). Proteomic analyses indicate that these granules contain hundreds of different types of proteins (Coppinger et al., 2004; Piersma et al., 2009). Protein cargos found in α-granules include neuroactive peptides that are more typically associated with chromaffin cells, including tachykinins and enkephalins (Graham et al., 2004). Conversely, proteomic studies suggest that chromaffin large dense-core vesicles (LDCVs) contain several major constituents of α-granules that can act in the vasculature, including platelet basic protein precursor, TGF-β, collagen isoforms, and metalloproteases (Table 2) (Wegrzyn et al., 2010). As with chromaffin cells, the mechanisms by which proteins are packaged in platelet storage granules are incompletely understood.

**Table 2:** **Comparison of granule types contained in platelets and chromaffin cells**.

	α-Granules	Dense granules	LDCVs
**Diameter**	200–500 nm	150 nm	150–300 nm
**Number**	50–80 per platelet	3–8 per platelet	∼10,000 per cell
**Percentage of cell volume**	10	∼1	13.5
**Contents**	Integral membrane proteins (e.g., P-selectin, αIIbβ3, GPIbα) Coagulants/anticoagulants and fibrinolytic proteins (e.g., factor V, factor IX, plasminogen) Adhesion proteins (e.g., fibrinogen, vWF) Chemokines [e.g., CXCL4 (PF4), CXCL12 (SDF-1α)] Growth factors (e.g., EGF, IGF) Angiogenic factors/inhibitors (e.g., VEGF, PDGF, angiostatins) Immune mediators (e.g., IgG, complement precursors)	Cations (e.g., Ca^2+^, Mg^2+^) Polyphosphates Bioactive amines (e.g., serotonin, histamine) Nucleotides (e.g., ADP, ATP)	Structural proteins (e.g., granins, glycoproteins) Vasoregulators (e.g., catecholamines, vasostatins, renin-angiotensin) Paracrine signaling factors (e.g., guanylin, neurotensin, chromogranin B) Immune mediators (e.g., enkelytin, ubiquitin) Opioids (e.g., enkephalins, endorphins) Ions (e.g., Ca^2+^, Na^+^, Cl^−^) Nucleotides (e.g., AMP, GDP, UTP) Nucleotides Polyphosphates

Platelet α-granule cargos can include coagulants and anticoagulants, angiogenic and antiangiogenic factors, proteases and proteases inhibitors, and proinflammatory and anti-inflammatory mediators. This observation has raised the question of how α-granules are able to efficiently mediate their biological functions when they contain so many proteins with opposing functions (Italiano et al., 2008; Blair and Flaumenhaft, 2009). One possibility is that there are different α-granule subpopulations that store distinct cargo. However, the number of discrete types of α-granule is not known. Evidence that α-granules are heterogeneous comes from several sources. Immunofluorescence microscopy demonstrated that the two α-granule cargos von Willebrand factor and fibrinogen do not localize to the same granule (Sehgal and Storrie, 2007). Subsequent studies showed that angiogenic factors localize to distinct compartments and were differentially released by different agonists (Italiano et al., 2008). The molecular mechanisms that mediate differential release are unclear. Differential distribution of SNAREs among subpopulations of α-granules may account for differential release. For example, Peters et al. (2012) showed that a population of granules containing vesicle-associated membrane protein-7 (VAMP-7) physically separated from VAMP-3 and VAMP-8-containing granules during spreading. However, the idea of α-granule heterogeneity remains controversial and some investigators in the field believe that granule cargos are stochastically distributed and that differential release either does not occur or is controlled at the level of pore expansion.

Granule heterogeneity and differential release have also been evaluated in chromaffin cells. Morphologic studies demonstrate heterogeneity among both LDCVs and synaptic-like microvesicles (SLMVs) (Koval et al., 2001). Studies using carbon-fiber amperometry to measure catecholamine release from individual granules indicated distinct granule populations on the basis of release kinetics (Tang et al., 2005). Different SNAREs and SNARE chaperones may associate with different granule populations and facilitate differential release. For example, different synaptotagmin isoforms associated with LDCVs and SLMVs and this observation could account for their differential secretion in response to calcium (Matsuoka et al., 2011). Other factors influencing chromaffin granule release include pore expansion kinetics and degree. Basal levels of catecholamine release may occur through a restricted fusion pore, while in response to excitation dynamin and myosin-mediated mechanisms may elicit fusion pore expansion (Chan et al., 2010). In addition, large aggregates of chromogranin A require complete fusion to facilitate release (Perrais et al., 2004; Felmy, 2007).

### Dense granules

Dense granules are a subtype of lysosome-related organelle (LRO). There are ∼3–6 dense granules/platelet (Flaumenhaft, 2013). These granules are so electron dense that they can be detected by whole mount electron microscopy in the absence of staining. They are highly osmophilic when viewed by TEM. Dense granules play a critical role in hemostasis and thrombosis, releasing factors such as ADP and epinephrine that act in an autocrine and paracrine manner to stimulate platelets at sites of vascular injury. Dense granules also contain factors that are vasoconstrictive such as serotonin (Flaumenhaft, 2013).

**Figure 2:**
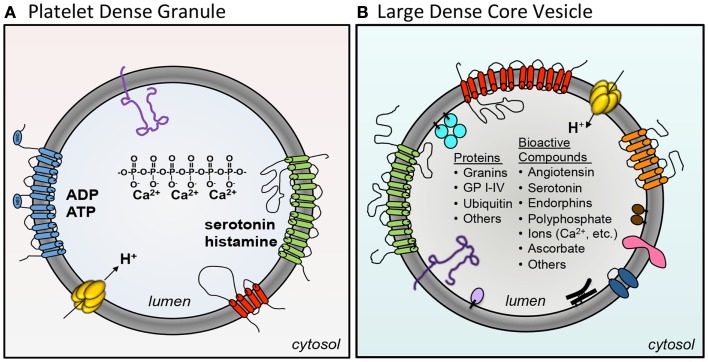
**A comparison of platelet dense granules and chromaffin LDCVs**. **(A)** Several membrane pumps concentration granule contents in the maturing granule. VMAT2 concentrates serotonin (*green*). An H^+^-ATPase proton pump maintains the granule at pH ∼5.4 (*yellow*). MRP4 (*blue*) is thought to concentrate adenine nucleotides into dense granules. Dense granules also express the tetraspanin CD63 (*red*) and the lysosomal marker LAMP-2 (*purple*). Dense granules contain a core of calcium chelated by polyphosphate. **(B)** The chromaffin large dense-core vesicle (LDCV) express a variety of membrane proteins including VMAT1 amine transporter (*red*), H^+^-ATPase (*yellow*), Cytochrome b561 (*orange*), p65 (*pink*), peptidyl α-amidation monooxygenase (PAM) (*blue*), LAMP-1 (*dark purple*), and VNUT/Slc17a nucleotide carrier (*green*). In addition, the following peripheral proteins are associated with the LDCV membrane: endopeptidases PC1/PC2 (*brown*), GPIII/SGP2/clusterin (*black*), carboxypeptidase H (*lavender*), and Dopamine β-hydroxylase (DβH) (*turquoise*). The LDCV core contains a large number of different proteins and bioactive compounds.

Dense granules and LDCVs have been compared based on their unusually high concentrations of cations, polyphosphates, adenine nucleotides, and bioactive amines such as serotonin and histamine (Sigel and Corfu, 1996) (Figure 2; Table 2). In platelets, adenine nucleotides are concentrated at ∼653 mM ADP and ∼436 mM ATP (Holmsen and Weiss, 1979). Calcium is at 2.2 M. Chromaffin granules and platelet dense granules are among the few mammalian granule types to contain polyphosphates (Aikawa et al., 1971; Ruiz et al., 2004). Active transport mechanisms are thought to contribute to efficient concentration of these constituents in platelets (Figure 2). A vesicular H^+^-ATPase proton pump maintains the dense-granule lumen at pH ∼5.4 (Dean et al., 1984), similar to the pH of LDCVs. The multidrug transporter MRP4, a multidrug resistance protein, is found on platelet dense granules and is proposed to transport adenine nucleotides into these granules (Jedlitschky et al., 2004). Uptake of serotonin from platelet cytosol into dense granules is mediated by vesicular monoamine transporter 2 (VMAT2). Transport is driven by an electrochemical proton gradient across the granule membrane. VMAT2 also appears to mediate histamine transport into dense granules (Fukami et al., 1984). The primary nucleotide transporter in chromaffin cells is Slc17A/VNUT (Sawada et al., 2008). Whether or not platelets use Slc17 family transporters to concentrate dense-granule cargo has yet to be evaluated. Like platelets, chromaffin cells use VMAT2, in addition to VMAT1, to pump monoamines from the cytosol into their granules.

### Lysosomes

Platelets contain few primary and secondary lysosomes. These lysosomes contain many acid hydrolases and cathepsins as cargo and express CD63 and LAMP-2 in their membrane. Platelet lysosome function is not well-studied. They may serve a role in endosomal digestion, as observed in nucleated cells (Flaumenhaft, 2013).

## An Overview of Platelet Granule Release

Platelets are uniform discoid cells that circulate in a quiescent state and undergo a dramatic morphological change when activated. Their plasma membrane surface area is ∼19 μm^2^ and the total surface area of their granules is ∼14 μm^2^. They have an unusual membrane system, including an open canalicular system (OCS), which is a system of tunneling invaginations of the plasma membrane that is unique to platelets and is estimated to have a surface area of ∼14 μm^2^ (Flaumenhaft, 2013). The OCS tracks through the platelet, but is topologically similar to the plasma membrane in that it possesses both an extracellular and a cytosolic face. Platelets also have a dense tubular system (DTS), which is a membrane system thought to be derived from the megakaryocytic endoplasmic reticulum. The DTS serves as an intracellular calcium storage site, but is not directly connected to either the plasma membrane or the OCS (van Nispen tot Pannerden et al., 2010).

Ultrastructural studies have demonstrated several atypical features of the platelet release reaction. In the resting state, platelet α-granules and dense granules are distributed throughout the platelet. With activation-induced shape change, granules become localized in a central granulomere. As with chromaffin granules, platelet granules may fuse with one another in a process termed homotypic fusion (Ginsberg et al., 1980). However, during exocytosis platelet granules then fuse with the OCS (Stenberg et al., 1984; Escolar and White, 1991). Granule contents are released into the OCS and diffuse out into the extracellular environment (Escolar and White, 1991). Exocytosis via fusion directly with plasmalemma has also been described (Morgenstern et al., 1987). SNAREs are localized on platelet membranes in a manner to support fusion of granules with OCS, plasma membrane, or other granules (Feng et al., 2002). Despite the morphological differences between exocytosis in platelets and chromaffin cells, similarities in the release mechanism have enabled platelet biologists to use chromaffin cells as a model in studying platelet granule release. For example, both platelets and chromaffin cells require Ca^2+^ influx as a mediator of exocytosis via different mechanisms. Upon platelet activation by agonists, the concentration of cytosolic Ca^2+^ increases activating protein kinase c (PKC), which is important for granule secretion (Knight et al., 1988; Flaumenhaft, 2013). Formation of an action potential in chromaffin cells triggers Ca^2+^ influx via Ca^2+^ channels thereby triggering exocytosis (Knight and Scrutton, 1980; Knight et al., 1982; Knight and Baker, 1985; Penner and Nicher, 1988; Cheek and Barry, 1993; Livett, 1993; Aunis, 1998; Garcia et al., 2006).

## The Cytoskeletal as Both Barrier and Facilitator in Exocytosis

The observation that platelet granule secretion occurs concurrently with a dramatic change in the shape of the platelet has prompted investigators to evaluate the role of the cytoskeleton in granule release. Platelets are rich in actin, which is the most abundant platelet protein. The resting platelet contains 40% filamentous actin (F-actin). Upon platelet activation, the percentage of F-actin increases to 80%. Studies using cytochalasins (Cox, 1988), latrunculin A (Flaumenhaft et al., 2005), Ca^2+^-mediated stimulation of the F-actin severing protein scinderin (Marcu et al., 1996), and PKC-mediated stimulation of MARCKS (Trifaro et al., 2002) demonstrate increased dense-granule release with inhibition of actin polymerization or with cleavage of F-actin. Inhibition of actin polymerization also augments the kinetics and degree of α-granule release (Flaumenhaft et al., 2005). These results suggest that F-actin disassembly might actually be required for normal granule secretion and that activation-mediated granule release is related to actin.

In contrast to the barrier function that the cytoskeleton serves in the resting state, *de novo* actin polymerization during platelet activation contributes to granule release as evidenced by the observation that high concentrations of inhibitors of actin polymerization block α-granule release (Woronowicz et al., 2010). These studies led to speculation that an actin barrier helps prevent inappropriate α-granule exocytosis, but that some *de novo* actin polymerization is required for α-granule release. Woronowicz et al. (2010) demonstrated that the target membrane SNARE (t-SNARE) SNAP-23 associates with the actin cytoskeleton of resting and activated platelets. In a cell-free platelet granule secretory system, inhibition of F-actin formation blocks release of SNARE-dependent α-granule contents, whereas actin polymerization stimulates α-granule release (Woronowicz et al., 2010). Yet the molecular mechanism by which the binding of SNAREs to the platelet cytoskeleton facilitates granule release is unknown. Overall, actin polymerization appears to serve a bipartite role in platelet granule secretion, both as a barrier to prevent inadvertent loss of thrombogenic cargo and as a facilitator of secretion.

Actin has been shown to serve a barrier function in chromaffin cells. The most well-studied pathways for disrupting the cortical F-actin barrier during chromaffin exocytosis include Ca^2+^-mediated stimulation of scinderin and PKC-mediated stimulation of MARCKS (Trifaro et al., 2002). Scinderin also potentiates Ca^2+^-induced granule secretion in a permeabilized platelet system and inhibitory peptides directed at scinderin inhibited granule release in this same assay (Marcu et al., 1996). MARCKS-derived inhibitory peptides blocks phorbol ester-induced platelet granule release, invoking MARCKS phosphorylation and deactivation in facilitating the disruption of F-actin required for granule release (Elzagallaai et al., 2000, 2001).

## SNARE Function in Platelet and Chromaffin Granule Exocytosis

Soluble NSF attachment protein receptors, or SNAREs, assemble into complexes to form a universal membrane fusion apparatus (Jahn and Scheller, 2006). Although all cells use SNAREs for membrane fusion, different cells possess different SNARE isoforms. Neurons and neuroendocrine cells use a set of SNAREs that is distinct from those used in non-neuronal cells. In contrast, platelets and chromaffin cells use many of the same chaperone proteins to regulate SNARE-mediated secretion (Table 3).

**Table 3:** **SNAREs and SM proteins in platelets and chromaffin cells**.

	Platelets	Chromaffin cells
v-SNARES	Vamp-2	**VAMP-2**
	Vamp-3	VAMP-3
	Vamp-4	VAMP-7 (TI-VAMP)
	Vamp-5	
	Vamp-7 (TI-VAMP)	
	**Vamp-8**	
t-SNARES	**SNAP-23**	SNAP-23
	SNAP-25	**SNAP-25a**
	SNAP-29	**SNAP-25b**
	Syntaxin-1	**Syntaxin-1A**
	Syntaxin-2	**Syntaxin-1B**
	Syntaxin-4	Syntaxin-2
	Syntaxin-7	Syntaxin-3
	Syntaxin-8	Syntaxin-4
	**Syntaxin-11**	
	Syntaxin-12	
Munc13 family	**Munc13-4**	Munc13-1
		Munc13-4
Munc18 family	Munc18-1	**Munc18-1**
	**Munc18-2**	Munc18-2
	Munc18-3	Munc18-3

*Essential components of the secretory machinery are highlighted*.

*Criteria: platelets: Vamp-8, murine knockout; SNAP-23, inhibitory antibodies, inhibitory peptides, overexpression of dominant negative construct; syntaxin-11, FLH4; Munc13-4, murine knockout, FLH3; Munc18-2, FLH5*.

*Chromaffin: VAMP-2, neurotoxin cleavage; SNAP-25, deletion of C terminus; Syntaxin 1, botulinum neurotoxin C1 and inhibitory antibodies; Munc18-1, murine knockout*.

VAMP-8 (endobrevin) is the primary and most abundant vesicular SNARE (v-SNARE) in platelets (Ren et al., 2007; Graham et al., 2009). It is required for activation-induced release of α-granules, dense granules, and lysosomes (Ren et al., 2007) as evidenced by studies using permeabilized human platelets exposed to anti-VAMP-8 antibodies and by evaluation of secretion from VAMP-8^−*/*−^ platelets (Ren et al., 2007). Platelet-mediated thrombus formation relies on ADP and other factors released from platelet granules. VAMP-8^−*/*−^ mice demonstrate decreased thrombus formation upon vascular injury (Graham et al., 2009). Electron microscopy indicates that platelet VAMPs localize primarily to granule membranes (Feng et al., 2002). VAMP-2, -3, -5, and -7 are also present in platelets. VAMPs 2 and 3 mediate granule release in VAMP-8 deficiency (Ren et al., 2007). VAMP-7 contains a profilin-like longin domain, has been shown to function in neurite extension, and associates with F-actin during cell spreading (Alberts et al., 2006). Granules expressing VAMP-7 move to the periphery of the platelet during spreading and may represent a distinct granule type that functions to provide membrane to cover growing cytoskeletal structures following activation (Peters et al., 2012). Future studies will evaluate the respective roles of VAMP-8 and VAMP-7 in mediating granule release during spreading and identify the participating membrane compartments.

Synaptosomal-associated protein 23 (SNAP-23), a t-SNARE, is required for release from all three types of granules in platelets (Chen et al., 2000; Lemons et al., 2000). Nearly 2/3rds of SNAP-23 associates with the platelet plasma membrane, with the remaining SNAP-23 distributed between the granule membrane and membranes of the OCS (Feng et al., 2002). SNAP-23 contains five palmitoylation sites in its membrane-binding domain. Cleavage of palmitate by acyl-protein thioesterase 1 releases SNAP-23 from platelet membranes demonstrating that SNAP-23 associates with membranes via these palmitoylation sites (Sim et al., 2007). In addition, SNAP-23 associates with the actin cytoskeleton in both resting and activated platelets (Woronowicz et al., 2010). Antibodies to SNAP-23 or addition of an inhibitory C-terminal peptide against SNAP-23 both block dense-granule release in human platelets (Chen et al., 2000). In addition, overexpression of dominant negative SNAP-23 inhibits dense-granule release in murine platelets (Gillitzer et al., 2008).

Our understanding of the role of syntaxins, another family of t-SNAREs, in platelet granule release has recently evolved. Platelets express syntaxin-2, -4, -7, -8, -11, and -12. Whiteheart’s group identified a patient with Familial Hemophagocytic Lymphohistiocytosis type four (FHL-4) who was deficient in syntaxin-11 and exhibited a significant granule secretion defect. An inhibitory antibody that this group had previously used to demonstrate a role for syntaxin-2 in granule release was found to cross-react with syntaxin-11, further suggesting a role for syntaxin-11 in platelet exocytosis (Ye et al., 2012). They also demonstrated that syntaxin-2^−*/*−^ mice, syntaxin-4^−*/*−^ mice, and double knockout mice all demonstrated normal granule release. On the basis of these results, syntaxin-11 appears to be the primary syntaxin involved in platelet granule release.

In chromaffin cells, VAMP-2 is the primary v-SNARE and is required for efficient, rapid release of granule constituents in response to agonists (Table 3). Proteolytic cleavage of VAMP-2 by botulinum neurotoxins A through G or tetanus neurotoxin results in decreased DCV secretion in chromaffin cells (Knight et al., 1985; Schiavo et al., 1992; Xu et al., 1998). VAMP-3 is less efficient than and plays a subordinate role to VAMP-2, only functioning in its absence (Borisovska et al., 2005). VAMP-7 serves a central role in neurite outgrowth in chromaffin-like cells (Coco et al., 1999; Martinez-Arca et al., 2000, 2001), analogous to its putative role in providing membrane for platelet spreading (Peters et al., 2012). Studies in PC12 cells indicate that the NH2-terminal domain of VAMP-7 negatively regulates neurite outgrowth since neurite outgrowth is blocked by overexpression of this domain and enhanced by its deletion (Martinez-Arca et al., 2000, 2001). SNAP-25a has established roles in both docking and priming of DCVs and participates in agonist-dependent fusion of secretory vesicles in complex with both VAMP-2 and syntaxin-1A. Deletion of the C-terminal synaptotagmin-interacting residues of SNAP-25 in PC12 cells results in decreased DCV secretion (Zhang et al., 2002). Adrenal chromaffin cells express syntaxins-1A, -1B, -2, -3, and -4. Viral infection with botulinum neurotoxin C1 cleaves syntaxin-1A, 1B, 2, and 3 resulting in reduced DCV docking at the plasma membrane (de Wit et al., 2006) and an inhibitory antibody to syntaxin-1 decreases catecholamine release in bovine chromaffin cells (Gutierrez et al., 1995).

## SNARE Chaperone Function in Platelet and Chromaffin Granule Exocytosis

**Figure 3:**
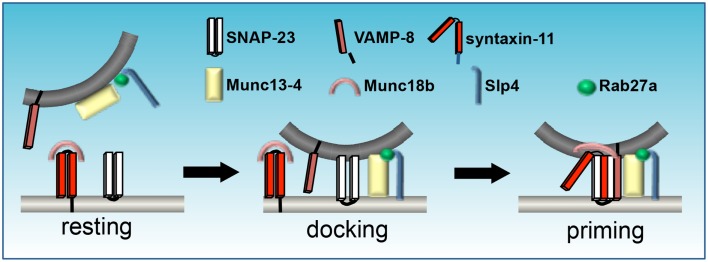
**Assemblage of SNAREs and SM proteins during platelet granule exocytosis**. Munc18b sequesters syntaxin in an inactive state. Munc13-4 docks opposing membranes via interactions with Rab27a, which also binds Slp1. Activation promotes a conformational change in Munc18b that enables the coiled-coil domain of syntaxin to form a four-helical bundle with SNAP-23 and VAMP. Mutations in Munc13-4, as in familial hemophagocytic lymphohistiocytosis (FHL)-3, syntaxin-11 (FHL-4), Munc18b (FHL-5), or Rab27a (Griscelli syndrome) result in defective secretion (figure adapted from Flaumenhaft, 2013).

Although not members of the exocytic core complex, another important group of proteins involved in degranulation in secretory cells are the Sec1/Munc18-like (SM) proteins which function as SNARE chaperones (Carr and Rizo, 2010). Platelets and chromaffin cells possess a similar repertoire of SM proteins, including members of the Munc13 and Munc18 families. These proteins are SNARE regulators that have no apparent membrane-binding domain, but bind syntaxin upon phosphorylation by PKC (Houng et al., 2003; Schraw et al., 2003) and interact with the regulatory N-terminal sequence of syntaxins (Ashery et al., 2000; Rosenmund et al., 2002). Munc13 family members include Muncs13-1, -2, -3, and 4. These proteins have two C2 (Ca^2+^-binding) and one C1 (DAG/phorbol ester-binding) domains (Figure 3). They interact with SNARE proteins via two Munc13 (mammalian) homology domains, MHDs 1 and 2 (Guan et al., 2008), which are involved in dissociating Munc18 protein/syntaxin interactions (Sassa et al., 1999), thereby promoting *trans* SNARE complex assembly.

Munc13-4 is the only Munc13 family member found in platelets. It lacks the N-terminal C1 domain present in Munc13-1, -2, and -3 and the MHD2 domain present in the other Munc13 family members, but has a central MHD1 domain and binds directly to syntaxins in platelets via interaction with the syntaxin H3 domain (Boswell et al., 2012). It is ubiquitously expressed, but enriched in cells of the hematopoietic lineage (Song et al., 1998; Feldmann et al., 2003). In platelets, the Munc13-4 interaction with activated Rab27a/b is important for SNARE binding (via MHD1 interaction), granule formation and plasma membrane interaction (Figure 3) (Song et al., 1998; Shirakawa et al., 2004; Ishii et al., 2005; Boswell et al., 2012). Boswell et al. (2012) determined that the C2A domain of Munc13-4 is required for Ca^2+^-dependent SNARE interaction, whereas the C2B domain mediates Ca^2+^-dependent membrane association. Mutation of Munc13-4 results in another form of familial hemophagocytic lymphohistiocytosis (FHL3) (Feldmann et al., 2003) and Munc13-4 deletion from murine platelets results in complete ablation of dense-granule release and impaired release from α-granules *in vitro* indicating its importance in Ca^2+^ regulation of SNARE interactions with the plasma membrane (Ren et al., 2010).

Munc13-4 is a rate-limiting protein for granule exocytosis in both platelets and chromaffin cells. As with platelet granule release, Munc13-4 triggers rapid and efficient release of catecholamines from chromaffin-like PC12 cells (Boswell et al., 2012). Munc13-4 promotes trans-exocytic core complex formation in a Ca^2+^-dependent manner in both chromaffin cells and platelets. In addition to Munc13-4, Munc13-1 serves a role in DCV secretion in chromaffin cells. Overexpression of Munc13-1 results in increased DCV secretion (Ashery et al., 2000; Stevens et al., 2005) and its interaction with syntaxin-1 is important for DCV priming (Stevens et al., 2005).

Platelets express three Munc18 isoforms: Munc18-1, 18-2 and 18-3. All three isoforms are associated with granule and OCS membranes in resting platelets (Schraw et al., 2003). Al Hawas et al. (2012) recently demonstrated that defects in the Munc18-2 gene result in familial hemophagocytic lymphohistiocytosis type 5 (FHL5). These patients demonstrate decreased α- and dense-granule secretion and levels of both Munc18-2 and syntaxin-11 were diminished, indicating that Munc18-2 plays a key role in platelet exocytosis and, potentially, a regulatory role toward syntaxin-11.

In chromaffin cells, Munc18-1 participates in granule docking/priming and SNARE engagement via its interaction with syntaxin 1 (Hata et al., 1993; Pevsner et al., 1994). Munc18-1 knock out in embryonic cells results in decreased DCV docking at the plasma membrane (Voets et al., 2001; Gulyas-Kovacs et al., 2007). In addition, syntaxin 1 expression is decreased by 50% in Munc18-1 deficient neurons and chromaffin cells (Voets et al., 2001; Gulyas-Kovacs et al., 2007). Munc18-1 interaction with the “closed” conformation of syntaxin 1 appears to be important for docking of the secretory vesicle at the plasma membrane (Dulubova et al., 1999; Yang et al., 2000; Schutz et al., 2005; Gulyas-Kovacs et al., 2007). However, Munc18-1 interactions with the N-terminal peptide of syntaxin-1 (in the “open” conformation) is required for membrane fusion to occur (Khvotchev et al., 2007; Gerber et al., 2008; Rathore et al., 2010), indicating that Munc18-1 is important in both early and late stages of exocytosis. Munc18-2 also shows affinity for syntaxins-1, -2, and -3 in chromaffin cells. While Munc18-2 rescued the reduced docking phenotype in Munc18-1^−*/*−^ animals, they continued to exhibit impaired vesicle priming (Gulyas-Kovacs et al., 2007). Munc18-3 is ubiquitously expressed and has been implicated in secretion in chromaffin cells. However, Munc18-3 only partially rescued the Munc18-1^−*/*−^ secretion defect in chromaffin cells and deletion of Munc18-3 from chromaffin cells did not cause defects in granule secretion (Gulyas-Kovacs et al., 2007).

## The Platelet Fusion Pore

**Figure 4:**
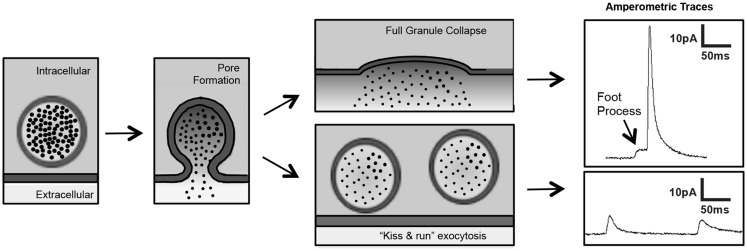
**Single-cell amperometry to measure dense-granule release from platelets**. Amperometry demonstrates pore formation progressing through a foot process (*left panels*) to full granule collapse (*upper panels*) and pore formation reversing in a “kiss and run” exocytotic event (*lower panels*) (figure adapted from Koseoglu et al., 2013).

Although there are many methods to evaluate platelet granule release, platelet secretion assays are largely restricted to bulk assays of cargo release (e.g., ADP, serotonin, platelet factor 4) or granule membrane receptor surface expression (e.g., P-selectin, CD63). These assays are inadequate for evaluation of the release of single granules and unable to detect membrane fusion events that occur in the millisecond time frame. Standard electrophysiology using patch-clamp techniques are difficult to apply to the platelet because of their small size and atypical membrane system. More recently, however, platelet investigators are applying some of the same approaches used to evaluate fusion pore dynamics in chromaffin cells. In particular, investigators are using single-cell amperometry to evaluate the release kinetics of single granules from platelets. Carbon-fiber microelectrode amperometry is being used to detect serotonin release from platelets stimulated with thrombin (Ge et al., 2008, 2009, 2010). Tracings indicate previously unrecognized fusion events such as “kiss and run” fusion and foot process formation (Wightman and Haynes, 2004) (Figure 4). This approach has enabled an appreciation of nuances of membrane fusion in platelets that have previously gone unrecognized and, more importantly, have enabled investigators to begin to evaluate the molecular mechanisms of pore formation in platelets. Amperometry has recently been used to evaluate the role of dynamin family proteins in platelet and chromaffin cell granule release.

Dynamins are a family of large GTPases that act as mechanoenzymes, demonstrating both oligomerization-dependent GTPase and membrane modeling activities (Piersma et al., 2009). Although originally described as mediators of membrane scission during vesicle endocytosis (Graham et al., 2004; Wegrzyn et al., 2010), dynamin GTPases are now recognized to function in exocytosis (Graham et al., 2002; Tsuboi et al., 2004; Fulop et al., 2008; Anantharam et al., 2010, 2011; Gonzalez-Jamett et al., 2010). In particular, dynamins act immediately upon membrane fusion to regulate the release of granule content. Dynamin and dynamin-related proteins are found in platelets. Dynamin 3 is upregulated during megakaryopoiesis (Reems et al., 2008; Gieger et al., 2011; Wang et al., 2011). Dynamin 2 and dynamin-related protein 1 (Drp1) are present in platelets, but dynamin 1 is not. Drp1 is phosphorylated upon platelet activation (Koseoglu et al., 2013). Inhibition of platelets using dynasore or MiTMAB, which inhibit the activity of dynamin family proteins, block agonist-induced platelet granule secretion (Koseoglu et al., 2013). The Drp1 inhibitor, mdivi-1, also blocks platelet granule exocytosis. Studies using single-cell amperometry demonstrate that mdivi-1 exposure results in fusion pore instability as evidenced by decreased foot process formation and inefficient pore expansion as evidenced by an increased T_1/2_ (Koseoglu et al., 2013). These observations implicate dynamin and dynamin-related proteins in platelet fusion pore dynamics. However, the mechanism by which Drp1, which is typically associated with mitochondrial fission, impacts platelet granule release remains to be determined.

Dynamin-mediated pore expansion in chromaffin has been evaluated using total internal reflection fluorescence microscopy and amperometry. In chromaffin cells overexpressing a dynamin I mutant with low GTPase activity, deformations in the membrane associated with fusion are long-lived, indicating defective pore expansion (Anantharam et al., 2011). Chromaffin cells overexpressing a dynamin I mutant with enhanced GTPase activity demonstrate increased pore expansion. These observations have led to a model in which dynamin restricts fusion pore expansion until GTPase activity is stimulated. The higher the GTPase activity, the faster the expansion of the fusion pore (Gerber et al., 2008). Dynamins appear to associate with actin, SNAREs, and synaptotagmin family proteins to participate in fusion pore expansion (Chan et al., 2010; Gu et al., 2010; Anantharam et al., 2012). However, the importance of these associations is poorly understood.

## Conclusion

Some characteristics of regulated secretion shared between platelets and chromaffin cells are common to all secretory systems. However, these secretory systems also share some unusual features that, if not unique to these cells, are not universally observed among secretory systems. These special commonalities may provide avenues for researchers investigating these cells types to further define these secretory systems. For example, the unusual density of LDCVs and platelet dense granules and their ability to concentrate nucleotides, bioactive amines, and polyphosphates raises the possibility that may use similar transporters. Some similarities in transporters such as VMAT2 have already been described. Further probing could reveal further overlap (e.g., Scl17A transporters in platelet dense granules, multidrug resistance transporters in chromaffin cells, or yet undiscovered transporters that are common to both cells). The ability of chromaffin-like PC12 cells to use VAMP-7 for neurite outgrowth and platelets to use VAMP-7 during spreading speaks to potential underlying similarities between the molecular mechanisms of membrane utilization during shape change. The role of dynamin family proteins in exocytosis is an emerging area of interest in secretion biology and further studies of these two cell types may reveal how they use these mechanoenzymes to regulate fusion pore formation during exocytosis. Historically, the study of the chromaffin cell has advanced more quickly than that of the platelet and has helped direct how platelet biologists have approached the study of granule exocytosis. As the study of platelet exocytosis progresses, understanding this secretory system may help chromaffin cell biologists better understand elements of granule formation and exocytosis in neuroendocrine cells.

## Conflict of Interest Statement

The authors declare that the research was conducted in the absence of any commercial or financial relationships that could be construed as a potential conflict of interest.
